# Air pollution leaves a redox-proteomic imprint: From shared inflammation to disease-specific vulnerability

**DOI:** 10.1016/j.redox.2026.104471

**Published:** 2026-11-04

**Authors:** Parag Jain, Akanksha Jain

**Affiliations:** a School of Pharmacy, Rungta College of Pharmaceutical Sciences and Research, International Skills University, Bhilai, Chhattisgarh, 490024, India; b Swami Atmanand Govt English Medium Model College, Somni, Rajnandgaon, India

Dear Editor,

We read with great interest the study of Jiang et al. that offers an in-depth proteomic dissection of the molecular pathways linking ambient air pollutants to mortality and major chronic diseases. The study applies plasma proteomics, mediation analysis, Mendelian randomisation, colocalization, tissue/cell-type enrichment, and external validation, going beyond traditional exposure-outcome associations to provide a robust molecular basis for understanding how different chemical pollutants converge to influence human disease [1]. The identification of common immune-inflammatory proteins and pollutant and disease-specific pathways is particularly relevant to the redox biology of environmental injury.

Nevertheless, we believe the results support a broader interpretation that may be relevant to the field. Air pollution may establish a persistent “redox-proteomic imprint” that converts an initial oxidative insult into persistent immune, metabolic, vascular, and tissue-remodelling responses. We propose that redox imbalance might be an upstream biological state that connects apparently different disease phenotypes, rather than considering oxidative stress as one of several downstream consequences of pollution.

A first insight is given by the distinction between shared and pollutant-specific proteomic responses. Proteins associated with all four pollutants were mainly enriched in immune regulatory pathways whereas pollutant specific proteins were associated with vascular dysfunction, DNA damage, apoptosis and extracellular matrix remodelling. This pattern could suggest a potentially important two-stage model [2]. The conserved core of pollution-induced injury might be a common redox-immune response, while the subsequent disease-specific trajectory could depend on the physicochemical properties and tissue distribution of individual pollutants. This framework could account for how seemingly diverse exposures ultimately converge into overlapping systemic diseases that still retain distinct organ-level signatures.

The second and more interesting observation is the difference between environmental exposure and molecular representation of it. Exposure to PM10 itself was not significantly associated with mortality and the major chronic diseases examined, but the proteomic signature associated with PM10 was significantly associated with increased risks across outcomes. This finding suggests an intriguing possibility: circulating proteomic alterations may be a molecular amplifier or “biological memory” of exposure, more proximal to disease initiation than the exposure measurement itself [3], [4]. Thus, lack of a strong exposure–outcome association does not necessarily indicate biological inactivity; the relevant information may be encoded in coordinated changes in proteins involved in inflammation, oxidative stress, tissue injury and repair. This concept may be particularly useful in identifying individuals who have moved from an environmentally exposed state to a biologically susceptible state.

Third, more attention should be paid to convergence of oxidative-stress pathways across metabolic and renal disease. Air pollution is associated with pathways for the synthesis/recycling of glutathione and biological oxidation, which are associated with type 2 diabetes and chronic kidney disease. Importantly, this suggests redox buffering capacity as a shared susceptibility axis rather than a downstream disease marker. Therefore, individuals with low glutathione-dependent antioxidant capacity may be more susceptible to a given pollutant exposure, which may account for some of the heterogeneity in clinical responses [5]. Future studies that integrate longitudinal exposomics with glutathione redox couples, lipid peroxidation products, protein oxidation and proteomic signatures could test whether baseline redox reserve predicts subsequent development of disease.

A fourth insight relates to AGER; The discovery of AGER as a mediator enriched in alveolar type 1 cells and lung tissue, along with genetic evidence linking it to chronic respiratory disease and lung cancer, offers an interesting mechanistic bridge between oxidative injury, inflammation and tissue-specific pathology. AGER is thus a potential biomarker beyond just an indicator of disease. One hypothesis is that pollution-induced oxidative and carbonyl stress may increase ligand availability for the AGER axis, thereby converting an acute environmental insult into sustained pulmonary inflammatory signalling and tissue remodelling [6]. This possibility provides a testable avenue for future experimental studies to determine whether modulation of AGER signalling may interfere with the progression from pollutant-induced oxidative stress to chronic pulmonary inflammation and malignant transformation. Crucially, such a hypothesis needs to be proven experimentally and not just taken as a given from the current epidemiological data.

The study also shows an interesting hierarchy of molecular mediators. Proteins such as GDF15, IGFBP4, PIGR, SPINK1 and others were consistently mediating associations across several outcomes while proteins such as EPHA2, EFNA1, ANGPT2 and CEACAM5 showed more disease-specific patterns. This allows for the distinction between “core” pollution-response proteins and “branch-point” proteins that determine organ-specific disease progression [1], [7]. Such classification may have therapeutic importance; targeting the common redox-inflammatory core may reduce systemic biological injury, whereas intervention at disease-specific branch points could provide organ-selective protection without broadly suppressing physiological immune responses [8].

Finally, the combination of mediation analysis with Mendelian randomisation and colocalization provides an important foundation to move from descriptive environmental epidemiology to mechanism-oriented intervention. However, as the authors correctly point out, the MR results provide supportive, but not conclusive evidence of causality, as colocalization with a shared causal variant was only observed for 2 protein–outcome pairs. This distinction is particularly relevant in redox biology, where circulating proteins may represent both cause and consequence of oxidative injury. Future work should thus include repeated longitudinal proteomics measurements with direct redox phenotyping and tissue-specific validation.

These observations collectively support a conceptual model where air pollution induces a conserved redox–immune perturbation that is subsequently modulated by pollutant chemistry, tissue susceptibility, antioxidant reserve and disease-specific molecular networks. The resulting phenotypic proteome may therefore be an intermediate biological layer between environmental exposure and clinical disease. This view may help to convert pollution-associated proteomic signatures from passive biomarkers to mechanistically informed tools for population susceptibility identification and prioritisation of redox-, immune- or tissue-specific therapeutic interventions.

The study by Jiang et al. therefore offers more than a comprehensive catalogue of proteins associated with air pollution. An even deeper implication is that environmental exposure can be translated into disease via a measurable and potentially targetable redox-proteomic state. Once confirmed experimentally and longitudinally, this concept could represent a significant route for precision prevention of pollution-related chronic diseases.

## CRediT authorship contribution statement

**Parag Jain:** Conceptualization. **Akanksha Jain:** Writing – original draft.

## Funding source

No funding was received.

## Declaration of competing interest

The authors declare that they have no known competing financial interests or personal relationships that could have appeared to influence the work reported in this paper.

## Data Availability

Data will be made available on request.
